# Computational Study of Elastic, Structural, Electronic, and Optical Properties of GaMF_3_ (M = Be and Ge) Fluoroperovskites, Based on Density Functional Theory

**DOI:** 10.3390/molecules27165264

**Published:** 2022-08-18

**Authors:** Hukam Khan, Mohammad Sohail, Nasir Rahman, Rajwali Khan, Mudasser Hussain, Asad Ullah, Aurangzeb Khan, Abed Alataway, Ahmed Z. Dewidar, Hosam O. Elansary, Kowiyou Yessoufou

**Affiliations:** 1 Department of Physics, University of Lakki Marwat, Lakki Marwat 28420, Pakistan; 2 Department of Mathematical Sciences, University of Lakki Marwat, Lakki Marwat 28420, Pakistan; 3 Department of Physics, Abdul Wali Khan University, Mardan 23200, Pakistan; 4 Prince Sultan Bin Abdulaziz International Prize for Water Chair, Prince Sultan Institute for Environmental, Water and Desert Research, King Saud University, Riyadh 11451, Saudi Arabia; 5 Department of Agricultural Engineering, College of Food and Agriculture Sciences, King Saud University, Riyadh 11451, Saudi Arabia; 6 Plant Production Department, College of Food & Agriculture Sciences, King Saud University, Riyadh 11451, Saudi Arabia; 7 Floriculture, Ornamental Horticulture, and Garden Design Department, Faculty of Agriculture (El-Shatby), Alexandria University, Alexandria 21545, Egypt; 8 Department of Geography, Environmental Management, and Energy Studies, University of Johannesburg, APK Campus, Johannesburg 2006, South Africa

**Keywords:** density functional theory, fluoroperovskite, optical properties, structural properties, electronic properties

## Abstract

This paper explains our first-principle computational investigation regarding the structural, optical, elastic, and electrical characteristics of gallium-based GaMF_3_ (M = Be and Ge) perovskite-type (halide-perovskite) compounds. Our current computation is based on density functional theory (DFT) and is achieved with the help of the WIEN2k code. We used the Birch–Murnaghan equation for optimization; in both compounds, we found that both GaBeF_3_ and GaGeF_3_ compounds are structurally stable. For the computation of elastic characteristics, the IRelast package for calculating elastic constants (ECs) is utilized. These compounds are mechanically ductile, scratch-resistant, anisotropic, and mechanically stable, showing huge opposition to plastic strain. The modified Becke–Johnson (TB-mBJ) potential approximation method is used to calculate different physical characteristics and shows that GaGeF_3_ behaves as a metal, whereas the GaBeF_3_ compound is insulating in nature. The involvement of various electronic states in band structures is calculated using the theory of the density of states. The different optical properties of these compounds can be studied easily using their band gap energy. At high energy ranges, these substances demonstrate strong absorption. At low energies, the GaGeF_3_ compound is transparent, while the GaBeF_3_ compound is opaque to incoming photons. Investigation of the optical characteristics has led us to the conclusion that both GaGeF_3_ and GaBeF_3_ compounds can be used for high-frequency ultraviolet device applications. This computational work is considered to be the first time that we can study these compounds, which to our knowledge have not previously been experimentally validated.

## 1. Introduction

New perovskite compounds with improved characteristics are constantly being developed by materials scientists. Those compounds that have the chemical formula ABF_3_ generally have a fluoroperovskite structure. The atomic arrangement of this material was initially identified in the perovskite CaTiO_3_. The A cation has twelve halide atoms linked to it in the perovskite structure, whereas the B cation has six atoms of fluorine bonded to it. Fluoroperovskite compounds are a unique family of materials, with a stable crystalline structure and outstanding electrical characteristics that range from semiconductors to insulators. Because of their crucial relevance, radiation dosimeters, material scintillation techniques, and the semiconductor industry [1,2,3] can utilize them in lens materials for photonic lithography; for this reason, in recent years, these chemicals have received much attention. Many studies have examined the characteristics of perovskite compounds, with the majority concluding that they are mechanically stable and elastically anisotropic [4,5,6]. Photovoltaic features with large efficiency and good energy storage have widespread applications in cars, electronics, and lenses in the form of ABF_3_ composites [7,8,9]. Fluorine is combined with organic or inorganic elements, as well as transition metals (TM) to form stable fluoroperovskites. Fluoroperovskites with a wide band gap are the most widely applicable structures. These can be combined to make complex lattice-matched materials with huge bandgaps, allowing lattice matching and band gap engineering [1]. One common feature of these compounds is their broad energy band gap. These compounds have tremendous potential and offer low absorption edges; hence, in vacuum ultraviolet (VUV) and ultraviolet (UV) wavelengths they can be used as glass [10,11]. Recent work on fluoroperovskites has been reported in previous studies [12,13,14]. Harmel et al. [15] used DFT to explore some of the features of barium-based BaCsF_3_ fluoroperovskites, concluding that due to its broad direct band gap and its bands of the imaginary factor of the insulating features in the ultraviolet range, BaCsF_3_ will be suitable for optoelectronic devices. Daniel et al. [16] discussed a few of the characteristics of LiBaF_3_ and found that these chemical compounds are suitable for storing energy.

GaBeF_3_ is a new and significant class of ternary compounds; in terms of modern electrical technologies, these compounds have the potential to be used as a lens material. In the ultraviolet (UV) spectrum, compounds with band gaps that are bigger than 3.1 eV will perform better. The GaGeF_3_ compound demonstrates a metallic nature; it is a good candidate for electrical applications because it is an electrical conductor with high transparency over a narrow band of energies. The purpose of this study is to use DFT and the FP-LAPW technique to study the basic electronic, elastic, optical, and structural characteristics of GaMF_3_ (M = Ge and Be) fluoroperovskites, providing researchers with core data for future laboratory work on the above mentioned compounds.

## 2. Computational Methodology

The calculations for the compounds were carried out using the FP-LAPW technique [17,18,19,20,21], which is included in the WIEN2k Fortran simulation engine [22]. The electronic and other properties of the material, such as its optical properties and density of state, etc., were computed using the TB-mBJ method [23]. The exchange-correlation potential for structural and elastic features is managed using the generalized gradient approximation (GGA) [24]. To obtain a considerable degree of convergence, this study explores certain FP-LAPW base functions up to RMT (in the muffin-tin spheres, where RMT is the minimum radius) and is equal to 8, represented by K_max_ in our example, where K_max_ in the plane wave expansion shows the magnitude of the maximum, k. For GaMF_3_ (M = Ge and Be) compounds and F, the RMT radii in the muffin-tin sphere are 2.50, 1.50, and 1.70 atomic units (a.u). Within the muffin-tin spheres, the spherical harmonics were extended to l_max_ = 11, but the Fourier-expanded charge density was reduced to G_max_ = 13 (a.u). Within the energy range of 0.001 Ry, when the total energy is falling, the internally consistent field calculations are said to have converged. We obtained the physical parameters by placing the energy vs. volume curve using the state equation developed by Birch–Murnaghan [25]. The elastic constant for the cubic crystal symmetries was obtained using IRelast [26]; these were then utilized to examine the elastic properties. The dielectric function ε(ω) was also utilized to identify the optical characteristics [27,28].

## 3. Results and Discussion

This part of the paper contains a detailed scientific discussion of the outcomes produced using our proposed TB-MBJ methods. In this section, we shall explain the compounds’ structural and optical properties.

### 3.1. Structural Properties

**Figure 1:**
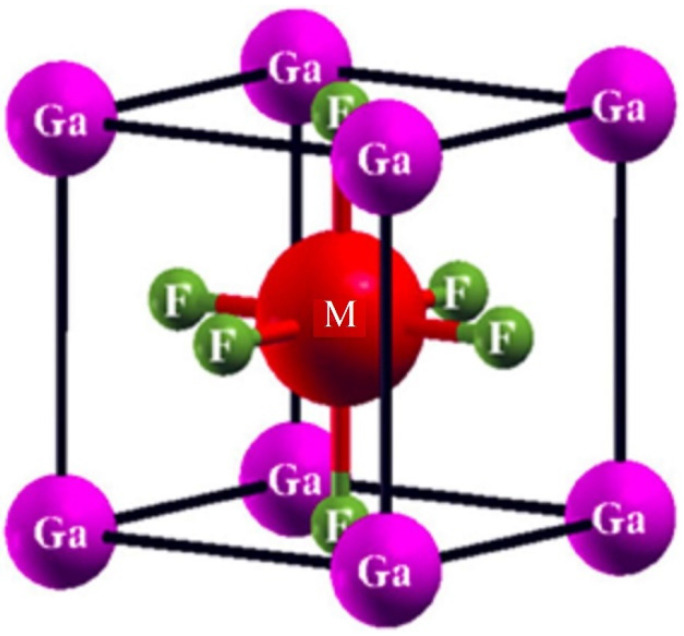
The prototypical crystal structure of the ternary compound, GaMF_3_ (M = Ge and Be).

**Figure 2:**
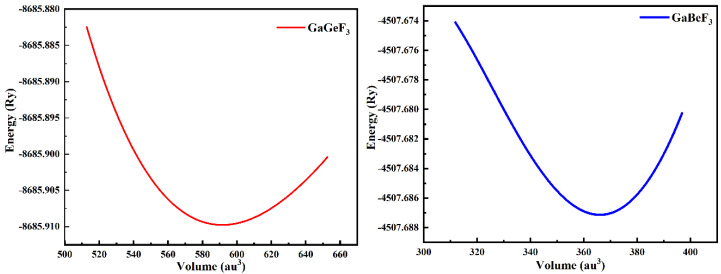
The optimized curve of GaMF_3_ (M = Ga and Be) compounds, fitted by Birch–Murnaghan’s equation of state.

GaMF_3_ (M = Ge and Be) crystallizes as a Pm3m (# 221) cubic perovskite structure, with one molecule as the unit cell. Ga atoms appear at (0,0,0), M atoms (M = Ge and Be) appear at (0.5,0.5,0.5), and F atoms appear at (0,0.5,0.5), (0.5,0,0.5), and (0.5,0.5,0), respectively. The Ga-based fluoroperovskite compound possesses a cubic structure, as shown in Figure 1. Around V_0_, we calculate the overall energy in terms of unit-cell volume (the cell volume in equilibrium conditions). The volume optimization approach can be used to forecast the structural features using the Birch–Murnaghan equation of state [25]. The Birch–Murnaghan equation is used to fit and establish ground state characteristics such as the lattice constant a_0_ at equilibrium and the bulk modulus B, as well as the derivative of pressure B′; thus, we performed analytical estimations of our obtained locations. Figure 2 shows the optimization curves, with the unit cell’s lowest-energy value versus the appropriate volume. The ideal or ground state E_0_ is the entire minimum energy value, versus volume. The volume is represented by V_0_, the optimum or ground state minimum volume. It is assumed that the structure of the compound with the highest optimum energy will be the most stable. Table 1 lists the ideal structural parameters that were determined, including a_0_ (optimized lattice constants), E_0_ (optimized ground state energy), B_0_ (bulk modulus), V_0_ (optimized volume), and B_0_ (bulk modulus pressure derivative). Because the bulk modulus decreases as the lattice constant increases, these results are in line with the overall trend of this approximation, suggesting that the calculated outcomes are more accurate and realistic. GaGeF_3_ has a steeper optimization fit curve than GaBeF_3_, indicating that GaBeF_3_ is structurally more stable. The above-mentioned Table 1 can also be used to compare structural stability.

### 3.2. Electronic Properties (Density of States and Energy Band Structures)

**Figure 3:**
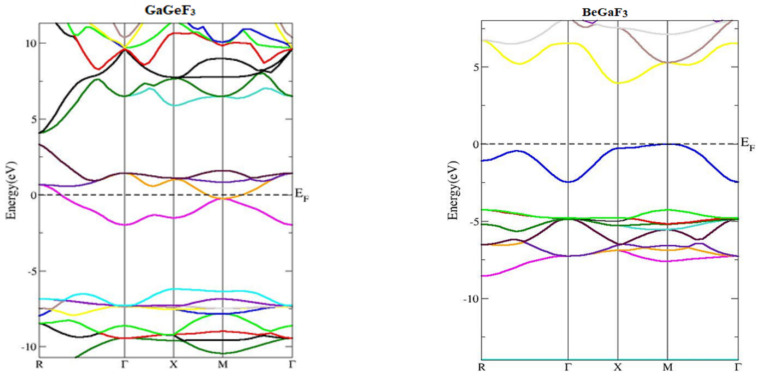
Energy band structures of compounds GaMF_3_ (M = Ge and Be), using TB-mBJ approximation.

**Figure 4:**
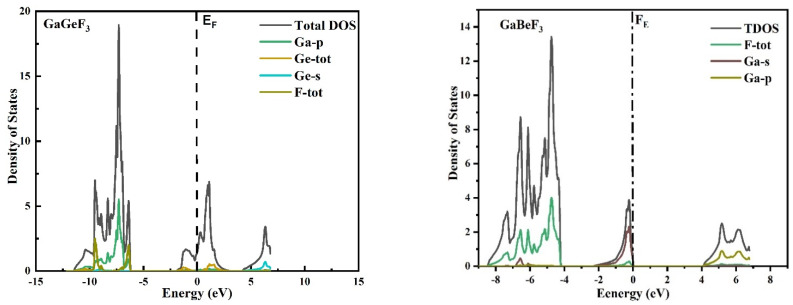
The TB-mBJ approximation approach was used to calculate the TDOS and PDOS of the GaMF_3_ (M = Ge and Be) compounds.

To investigate the electronic characteristics of GaMF_3_ (M = Ge and Be) compounds, we must determine the actual diagrams of band structures, their density of state (DOS), and the distribution of charges in this section. The LDA and GGA computations are well known for establishing the basic gaps of the bands of semiconductors and insulators [29,30]. The majority of this utility is due to their fundamental geometries failing to dependably reproduce both the exchange-correlation energy and its derivative of charge. To address this underestimating of band gaps, the modified Becke–Johnson potential (TB-Mbj) was adopted, and has been used successfully in a number of recent papers [14,31,32]. Figure 3 depicts the observed energy-band structures in the Brillouin zone for the geometry at the equilibrium of GaMF_3_ (M = Ge and Be) along high-symmetry directions. At the valence band top, the Fermi energy, EF, is chosen to be the zero-energy level. GaGeF_3_ is identified as metal because the valence band (VB) maxima and conduction band minima overlap. The valence band’s maxima occur at symmetry point M, while the conduction band’s minima occur at symmetry point X, yielding an indirect (X-M) energy gap of 3.89 eV for GaBeF_3_. To acquire a better understanding of the electronic structure, we have shown the TDOS and PDOS (total and partial atomic density of states) for GaMF_3_ (M = Ge and Be) compounds in Figure 4. DOS displays the contribution of several electronic states to the valence and conduction bands. The Fermi energy, EF, is shown by the vertical dashed lines at 0 eV, while the DOS spans −8 to 8 eV. The conduction band part of the DOS is to the right of EF, whereas the valance band is to the left.

The largest contributors to the DOS are F-tot and Ge-p; these represent states in the valence band with energies ranging from −6 to −11 eV and −2 eV to 0 eV, respectively, for GaGeF_3_. The largest contribution in the conduction band is from the Ga-p state for GaBeF_3_ in an energy range from 4 to 7 eV, whereas in GaBeF_3_, the largest contribution is from Ga-p and F-tot in the valance band, in an energy range from −4.2 to −8.3 eV. The conduction band of F-p and Ga-p has the largest contribution, as shown in Figure 4.

### 3.3. Elastic Properties

The elastic properties of the crystal can be calculated from the elastic constants in response to the external forces exerted on the compound. These constants’ measured values provide useful information about a compound’s toughness and stability. The elastic constants of the compounds were calculated at zero pressure by computing the stress tensor components for tiny deformation and applying energy, in line with a lattice deformation that maintained volume [33]. The IRelast package, which is integrated into WIEN2k and is specifically designed for cubic systems, was utilized to determine the elastic constants (Ecs). Because of the cubic crystal lattice symmetry, the three different elastic constants are C_11_, C_12_, and C_44_. These independent constants are summarized in Table 2. In order to have a mechanically stable cubic crystal structure, the following conditions of the elastic constants must be satisfied: C_11_ − C_12_ > 0, C_11_ > 0, C_44_ > 0, C_11_ + 2C_12_ > 0, and also B > 0 [34]. Here, the measured elastic constant Cij values reveal the elastic stability of our compounds. The C_11_ value for GaBeF_3_ is 93.4057 GPa, which is smaller than that of GaGeF_3_ at 98.525 GPa. Thus, GaGeF_3_ is somewhat harder than GaBeF_3._ The ability to form small cracks in materials is strongly linked to crystal A (elastic anisotropy), which can be implemented for specific purposes, especially in engineering research. From the supplied values of these elastic constants, we derived the A (anisotropy feature) to measure the elastic anisotropy of these materials, using the following equation:

A = 2C_44_/(C_11_ × C_12_) (1)

For an isotropic material, A equals 1, whereas any quantity less than 1 indicates anisotropy. Since the value of A fluctuates from 1, both of these materials are anisotropic, while the extent of the variant indicates the grade of anisotropy. From the computed data, as shown in Table 2, we know that this is −14.322 for GaBeF_3_, whereas those for GaGeF_3_ show that it is −0.40, indicating that GaGeF_3_ has a significant degree of anisotropy. The shear modulus, G, Young’s modulus, E, and Poisson ratio, *v*, must be obtained using elastic constants by the application of the following formulae [35,36,37]:

(2) $$E=\frac{9\mathrm{BG}}{G+3B}$$

(3) $$v=\frac{3B-2G}{2\left(G+2B\right)}$$

(4) $$G_{v}=\frac{C_{11}-C_{12}+3C_{44}}{5}$$

(5) $$G_{R}=\frac{5C_{44}\left(C_{11}-C_{12}\right)}{4C_{44}+3C_{11}-C_{12}}$$

(6) $$A=\frac{2C_{44}}{C_{11}-C_{12}}$$

The values of E, A, *v*, and G are computed from these equations and are shown in Table 2. To evaluate the level of ductility or brittleness of the material, a number of criteria can be used. The presence of ductility is shown as(C_11_ − C_44_), which is a Cauchy’s pressure that shows the change between C_11_ and C_44_ [38]. If the change between C_11_ and C_44_ is positive, the material exhibits ductility; if it has a negative value, the material exhibits brittleness. Here, Cauchy’s pressure for both materials is positive; that is, 24.75 GPa for GaBeF_3_ and 105.841 GPa for GaGeF_3_, indicating that both materials show ductility. The Pugh ratio, i.e., the B/G ratio, is another way to identify if a material is brittle or ductile. The limited value of the B/G ratio is 1.75 if a compound with a large Pugh ratio is supposed to be highly ductile [39]. Both compounds have different values from the critical point in this example, 13.29 for GaBeF_3_, and −25.127 for GaGeF_3_. As a result, GaGeF_3_ has somewhat higher ductility than GaBeF_3_. T. Frantsevich et al. [40] employed *v* (Poisson’s ratio) and reported a crucial value of 0.26 for discriminating between the ductility and brittleness of materials. Brittle materials have a value of less than 0.26, whereas ductile materials have a value greater than 0.26. As can be seen in Table 2, both ternary GaMF_3_ (M = Ge and Be) compounds have a higher value than 0.26, namely, 0.394 for GaBeF_3_ and 0.492 for GaGeF_3_, confirming their ductile character. In conclusion, we found that the compounds of GaMF_3_ (M = Ge and Be) are mechanically ductile, anisotropic, robust, and crack-resistant. We can certainly see multiple uses for these elastic characteristics in the future, in a variety of modern electronic technologies, based on these findings.

### 3.4. Optical Properties

We have exposed our material to light photons of energy starting at 14 eV; all the optical characteristics of both compounds are calculated using the expected lattice constant in equilibrium conditions. All optical characteristics may be found using the dielectric function ε(ω).

#### 3.4.1. The Dielectric Function

**Figure 5:**
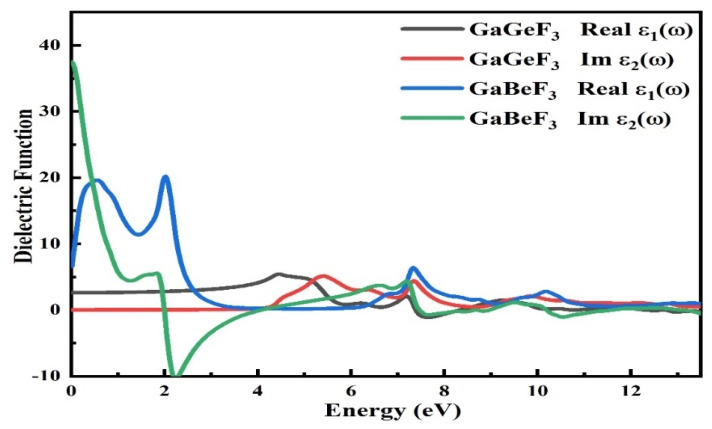
The calculated dielectric function ε(ω) for the GaMF_3_ compound (M = Ge and Be).

The dielectric function is represented mathematically by the symbol ε(ω), which includes the real component and imaginary component, which can be interpreted as ε(ω) = ε_1_(ω) + *i*ε_2_(ω). The measured real component is represented by ε_1_(ω) of the total dielectric function ε(ω), which disperses the incident light emitted by the material and electronically polarizes it, as shown in Figure 5. At zero electron volt energy, the value of the dielectric function for the compound GaGeF_3_ was found to be 2.5 and the highest peak value for the ε(ω) was 5 at 4.3 eV, while for the other compound, GaBeF_3_, the value was 2.6 at 0 eV and the greatest value was approximately 22 at 2.2 eV. At 1.2 eV, the value of ε_1_(ω) for GaBeF_3_ was also 20. According to the Penn model [41], the higher the dielectric function at zero energy ε_1_(0), the smaller the band gap energy, and vice versa.

The Penn model predicts that GaGeF_3_ has a ε_1_(0) value of 2.52, resulting in a large band gap of 5.131 eV, whereas GaBeF_3_ has a band gap of 5 eV. Within the energy range of 14.0 eV, the calculated ε_2_(ω) (the second component) of ε(ω) is shown in Figure 5. According to our inspection of the ε_2_(ω) spectrum, the first essential peak (critical energy) of the ε(ω) (dielectric permittivity) for the GaBeF_3_ and GaBeF_3_ takes place at around 6 eV. At the X-symmetry point, the absorption edge is responsible for a direct optical move from the valence to the conduction band. The curve begins to rise and fall over the threshold energy. GaGeF_3_ has a maximum peak of 5 at around 5.5 eV, while GaBeF_3_ has a maximum of 37.0 at 0.0 eV. Due to the direct broad band gap of the compound’s results, it is ideal for application in devices using ultraviolet light.

#### 3.4.2. The Refractive Index

**Figure 6:**
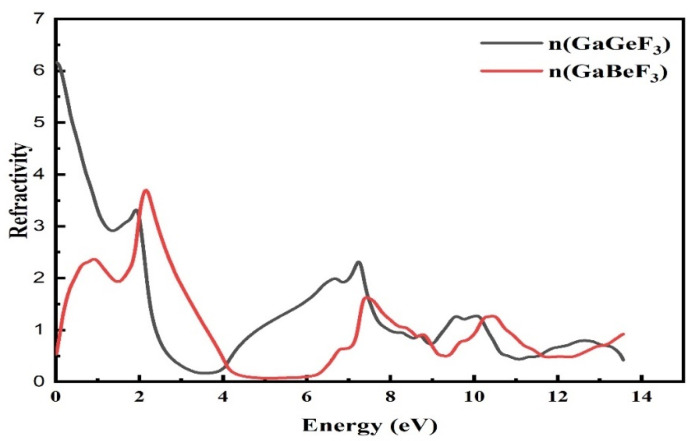
The calculated refractive index of the GaMF_3_ compound (M = Ge and Be).

To calculate different physical parameters, the refractive index is represented by η(ω), optical conductivity by σ(ω), the absorption coefficient by I(ω), and reflectivity, R(ω), using ε_1_(ω) and ε_2_(ω). Figure 6 depicts the calculated refractive index, η(ω). The static refractive index η(0) represents the refractive index at zero eV and it has a value of 0.5 and 6.2 for GaBeF_3_ and GaGeF_3_, respectively, as it is presented in the refractive index spectrum. It is obvious from Figure 6 that the curves of η(ω) for the compounds do not match and show little change. GaGeF_3_ has a maximum peak refractive index of 6.2 at 0 eV photon energy, while GaBeF_3_ has the highest peak refractive index of 3.81 at a photon energy of 2.21 eV, as shown in Figure 5. From the value of the refractive index, we are able to establish how much light is refracted from the material compound, which is especially useful in photoelectric applications. From Figure 6, it is clear that the photons are facing the obstacle as they enter the compound, due to the interaction of the photons with the electrons; this is why the refractive index is greater than one (η(ω) > 1). The larger a material’s refractive index, the more photons are deflected as they pass through it. Every procedure that increases a material’s density of electrons likewise enhances its refractive index.

#### 3.4.3. Absorption Coefficient

**Figure 7:**
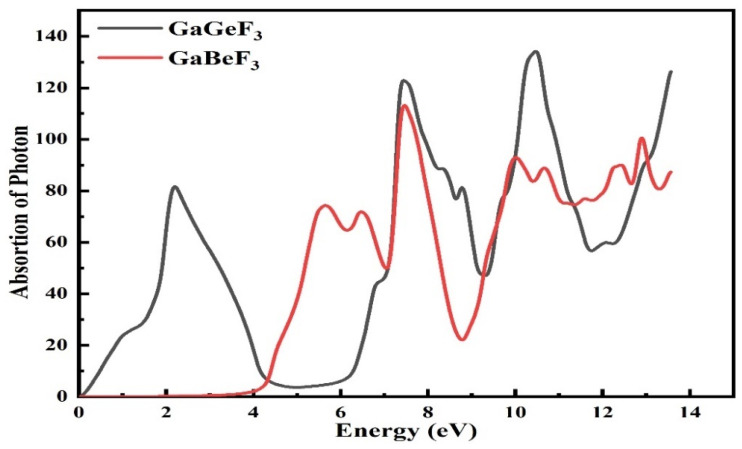
The calculated absorption coefficient of the GaMF_3_ compound (M = Ge and Be).

The absorption coefficient I(ω) can be obtained by the application of the dielectric function, ε(ω). The selected compounds have major absorption factors at energy levels ranging from 0 eV to 14.0 eV for GaGeF_3_, while GaBeF_3_ has energy levels ranging from 4.0 eV to 14.0 eV, as shown in Figure 7. This is the point at which a compound starts to efficiently absorb electromagnetic radiation. Both of these compounds have different threshold points, which are 0.0 eV and 3.80 eV for GaGeF_3_ and GaBeF_3_, respectively. Different absorption peaks of 81, 130, 138, and 122 occur for GaGeF_3_ at 2.20, 7.50, 10.30, and 13.70 eV of energy, respectively, whereas the absorption peaks of 72, 70, 118, 75, and 100 occur for GaBeF_3_ at 5.3, 6, 7, 10, 12, and 12.2 eV of energy, respectively.

#### 3.4.4. Reflectivity R(ω) Values

**Figure 8:**
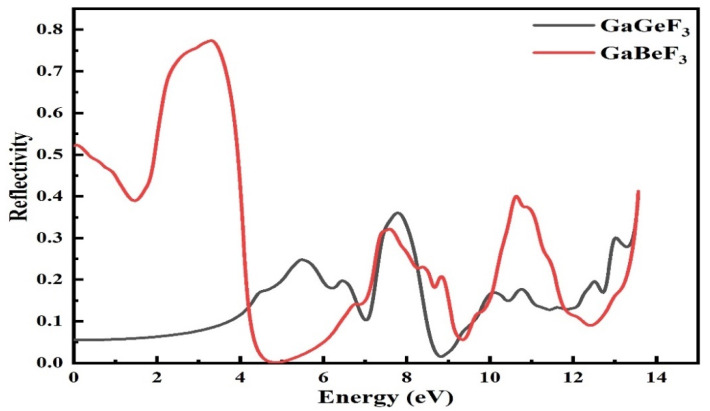
The computed reflectivity R(ω) of light from the GaMF_3_ compound (M = Ge and Be).

Figure 8 shows the R(ω) value, which is estimated from the dielectric permittivity, as shown throughout the range of energy of 0 eV to 14 eV. For GaGeF_3_ and GaBeF_3_, the reflectivity R(0) at zero-frequency is 0.05 and 0.55, respectively. As the photon energy increases, the reflectivity of GaBeF_3_ first decreases to 0.39 at 1.8 eV then rises to a maximum of 0.8 at 2.2 eV, then decreases to 0 at 4.48 eV, then increases, having different peaks of 0.3, 0.4, and 0.41 at 7.5, 10.5, and 13.5 eV, respectively. This is also the case for GaGeF_3_; as the photon energy increases, the reflectivity increases. The values are 0.05, 0.25, 0.35, and 0.34 at 0, 54, 7.8, and 13.5 eV, respectively. The reflectivity of GaBeF_3_ is very low compared to GaGeF_3_; hence, GeBeF_3_ is more transparent in the energy range from 0 to 4.4 eV than GaGeF_3_. The material’s transparency indicates that these compounds could be used to make lenses.

#### 3.4.5. Optical Conductivity

**Figure 9:**
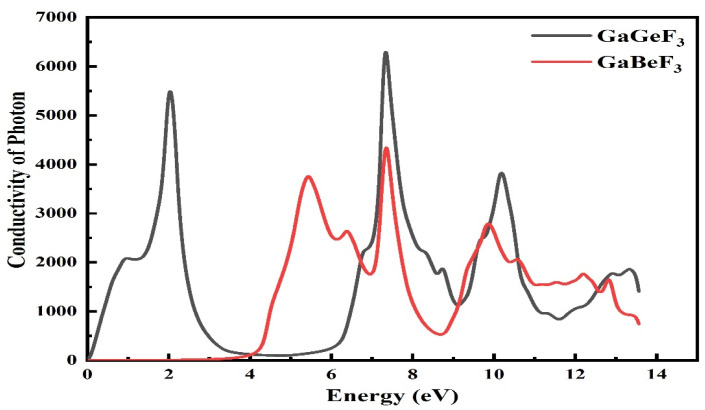
The computed conduction of incident light, represented by the σ(ω) of the GaMF_3_ (M = Ge and Be) compound.

The mathematical representation of photon conduction is σ(ω), which tells us the movement of electrons in a material, as caused by the application of an electromagnetic field. From the dielectric function, we can investigate the conductivity σ(ω), as depicted in Figure 9. The photon conductivity starts from 0 at 0 eV for the compound GaGeF_3_ and reaches a value of 5500 at 2.3 eV, then decreases to 100 in a range of energy of about 4.0 to 6.0 eV, reaching its maximum value of 6500 at 7.9 eV. Another peak of 4000 is observed at 10.3 eV, while for GeBeF_3_, the optical conductivity is nil at an energy range from 0 to 4 eV. Its value then increases, and peaks of 3500, 4500, and 3000 are observed at 5.1, 7.2, and 10 eV, respectively. As a result, we found that the compound of GaGeF_3_ was optically more conductive at low energy levels, compared to GeBeF_3_.

### 3.5. The Energy Loss Function (ELF)

**Figure 10:**
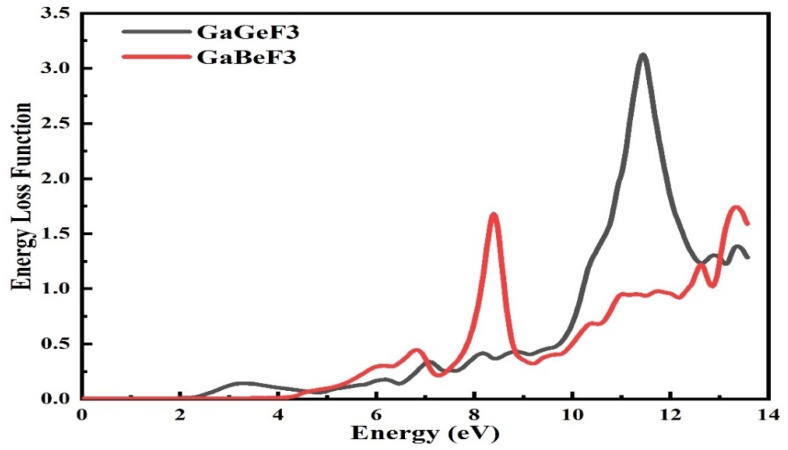
The computed optical energy loss function L(ω) of the GaMF_3_ (M = Ge and Be) compound.

To find or characterize the intra-band, inter-band, and Plasmon interdependencies’ energy loss function (ELF), the energy-loss function can be employed. When a fast-moving electron enters the material and passes through it, the electron decelerates and loses energy. Figure 10 shows the calculated optical energy loss function (ELF) for both compounds. Initially, there was minimum energy loss in the range of photon energies from zero eV and 2.4 eV, then a significant loss occurred from 5.0 eV to 14.0 eV. The highest energy loss, of 1.55 for GaBeF_3_ and 3.53 for GaGeF_3_, occurred with the energies of the incident photon at 8.8 eV and 12 eV, respectively.

## 4. Conclusions

In the current research, we successfully examined the structural, optical, electrical, and elastic properties of ternary fluoroperovskite GaMF_3_ (M = Ge and Be) compounds. These represent the most precise and creative results available. Based on optimal structural parameters, we came to the conclusion that GaMF_3_ (M = Ge and Be) compounds are cubic and structurally stable. To predict the elastic parameters, such as the fundamental elastic constant, the anisotropy factor, Poisson’s ratio, ductility, Cauchy’s pressure, shear modulus, Pugh ratio, and Young’s modulus, the IRelast package was used. These results give us confidence that these materials can be used in numerous contemporary electrical technologies. We investigated the fundamental electrical properties of the compounds of interest, using the TB-MBJ potential approximation method. GaGeF_3_ is a metal, while GaBeF_3_ is an insulator with an indirect (X-M) energy gap of 3.89 eV, as we have shown. The largest contributors to the DOS are F-tot and Ga-s, with states in the valence band for GaBeF_3_, and the largest contribution to the conduction band is from the Ga-p state for GaBeF_3_, whereas in GaGeF_3_, the largest contribution is from Ga-p and F-tot in the valance band, while in the conduction band, Ge-tot and Ge-s show the largest contribution.

## Figures and Tables

**Table 1:** Optimized crystal unit cell characteristics of the GaMF_3_ compound (M = Ge and Be).

Structural Specification	GaBeF_3_	GaGeF_3_
a_0_ (Å)	5.113	5.1035
V_0_ (a.u^3^)	891.00	897.01
B_0_ (Gpa)	33.20	33.79
B_0_/(Gpa)	4.7814	4.9494
E_0_ (Ry)	20,766.82	28,645.22

**Table 2:** For ternary GaMF_3_ (M = Ge and Be) compounds, the calculated elastic constants, bulk modulus, anisotropy factor, Young’s modulus, Poisson’s ratio, Pugh ratio (B/G), and Cauchy’s pressure are shown.

Elastic Parameters	GaGeF_3_	GaBeF_3_
C_11_(GPa)	98.525	93.4057
C_12_(GPa)	62.046	102.992
C_44_(GPa)	−7.316	68.648
B (GPa)	172.03	172.03
A	−0.40	−14.322
E (in GPa)	8.745	109.485
ʋ	0.492	0.394
B/G	−25.127	13.29
G (GPa)	−6.846	12.94

## Data Availability

The data presented in this study are available on request from the corresponding author.

## Sample Availability

Samples of the compounds not are available from the authors.
